# The RADx Tech Test Verification Core and the ACME POCT in the Evaluation of COVID-19 Testing Devices: A Model for Progress and Change

**DOI:** 10.1109/OJEMB.2021.3070825

**Published:** 2021-04-28

**Authors:** Eric J. Nehl, Stacy S. Heilman, David Ku, David S. Gottfried, Sarah Farmer, Robert Mannino, Erika Tyburski, Julie Sullivan, Allie Suessmith, Leda Bassit, Janet Figueroa, Anna Wood, Traci Leong, Anuradha Rao, Beverly Rogers, Robert Jerris, Sunita Park, Mark D. Gonzalez, Jennifer K. Frediani, Claudia R. Morris, Joshua M. Levy, Nils Schoof, Maud Mavigner, John D. Roback, Kristen Herzegh, Natia Saakadze, Jess Ingersoll, Narayana Cheedarla, Andrew Neish, Bradley Hanberry, Christopher C. Porter, Annette M. Esper, Russell R. Kempker, Paulina A. Rebolledo, Pamela D. McGuinness, Frederick Balagadde, Rebecca Gore, Ainat Koren, Nira Pollock, Eugene J. Rogers, Karl Simin, Nathaniel S. Hafer, Mary Ann Picard, Chiara E. Ghezzi, David D. McManus, Bryan O. Buchholz, Christina A. Rostad, Viviana Clavería, Thanuja Ramachandra, Yun F. Wang, CaDeidre Washington, Cheryl Stone, Mark Griffiths, Ray Schinazi, Ann Chahroudi, Miriam B. Vos, Oliver Brand, Greg S. Martin, Wilbur A. Lam

**Affiliations:** Behavioral, Social & Health Education Sciences, Emory University, Atlanta, GA 30322 USA.; Pediatrics, Emory University, Atlanta, GA 30322 USA.; School of Mechanical Engineering, Georgia Institute of Technology, Atlanta, GA 30332 USA.; Institute for Electronics and Nanotechnology, Georgia Institute of Technology, GA 30322 USA.; Georgia Institute of Technology, Atlanta, GA 30322 USA.; Department of Biomedical Engineering, Georgia Institute of Technology, Atlanta, GA 30322 USA.; The Atlanta Center for Microsystems-Engineered Point-of-Care Technologies, Georgia Institute of Technology, Atlanta, GA 30322 USA.; Pediatrics, Emory University, Atlanta, GA 30322 USA.; Laboratory of Biochemical Pharmacology, Emory University, Atlanta, GA 30322 USA.; Pediatrics, Emory University, Atlanta, GA 30322 USA.; Department of Pediatrics, Emory University School of Medicine, Atlanta, GA 30322 USA.; Department of Statistics, Emory University School of Public Health, Atlanta, GA 30322 USA.; Department of Pediatrics, Emory University School of Medicine, Atlanta, GA 30322 USA.; Department of Pathology and Laboratory Medicine, Emory University School of Medicine, Atlanta, GA 30322 USA.; Children’s Healthcare of Atlanta, Emory University School of Medicine, Atlanta, GA 30322 USA.; Emory University School of Public Health, Atlanta, GA 30322 USA.; Department of Pediatrics, Emory University School of Medicine, Atlanta, GA 30322 USA.; Department of Otolaryngology-Head & Neck Surgery, Emory University School of Medicine, Atlanta, GA 30322 USA.; Department of Pediatrics, Emory University School of Medicine, Atlanta, GA 30322 USA.; Department of Pathology and Laboratory Medicine, Emory University School of Medicine, Atlanta, GA 30322 USA.; Department of Pediatrics, Emory University School of Medicine, Atlanta, GA 30322 USA.; Department of Pathology and Laboratory Medicine, Emory University School of Medicine, Atlanta, GA 30322 USA.; Emory University School of Public Health, Atlanta, GA 30322 USA.; Department of Pathology and Laboratory Medicine, Emory University School of Medicine, Atlanta, GA 30322 USA.; Children’s Healthcare of Atlanta, Emory University School of Medicine, Atlanta, GA 30322 USA.; Department of Pediatrics, Emory University School of Medicine, Atlanta, GA 30322 USA.; Department of Medicine, Emory University School of Medicine, Atlanta, GA 30322 USA.; Medical Device Research, University of Massachusetts Lowell, Lowell, MA 01854 USA.; Department of Biomedical Engineering, University of Massachusetts Lowell, Lowell, MA 01854 USA.; School of Nursing, University of Massachusetts Lowell, Lowell, MA 01854 USA.; Department of Pathology, Boston Children’s Hospital, Boston, MA USA.; Department of Biomedical and Nutritional Sciences, University of Massachusetts Lowell, Lowell, MA 01854 USA.; Department of Molecular, Cell, and Cancer Biology, University of Massachusetts Medical School, Worcester, MA 01655 USA.; University of Massachusetts Medical School,Worcester, MA 01655 USA.; Medical Device Research, University of Massachusetts Lowell, Lowell, MA 01854 USA.; Department of Biomedical Engineering, University of Massachusetts Lowell, Lowell, MA 01854 USA.; Department of Medicine, UMass Medical School, Worcester, MA 01655 USA.; University of Massachusetts Lowell, Lowell, MA 01854 USA.; Department of Pediatrics, Emory University School of Medicine, Atlanta, GA 30322 USA.; School of Mechanical Engineering, Georgia Institute of Technology, Atlanta, GA 30332 USA.; Department of Medicine, Emory University School of Medicine, Atlanta, GA 30322 USA.; Department of Pathology and Laboratory Medicine, Emory University School of Medicine, Atlanta, GA 30322 USA.; Department of Medicine, Emory University School of Medicine, Atlanta, GA 30322 USA.; Children’s Healthcare of Atlanta Inc, Atlanta, GA 30322 USA.; Pediatric Emergency Medicine Unit, Emory University School of Medicine, Atlanta, GA 30322 USA.; Department of Pediatrics, Emory University School of Medicine, Atlanta, GA 30322 USA.; Department of Electrical and Computer Engineering, Georgia Institute of Technology, Atlanta, GA 30322 USA.; Department of Medicine, Emory University School of Medicine, Atlanta, GA 30322 USA.; Department of Pathology and Laboratory Medicine, Emory University School of Medicine, Atlanta, GA 30322 USA.

**Keywords:** COVID-19, Device Testing, RADx

## Abstract

Faced with the COVID-19 pandemic, the US system for developing and testing technologies was challenged in unparalleled ways. This article describes the multi-institutional, transdisciplinary team of the “RADx^SM^ Tech Test Verification Core” and its role in expediting evaluations of COVID-19 testing devices. Expertise related to aspects of diagnostic testing was coordinated to evaluate testing devices with the goal of significantly expanding the ability to mass screen Americans to preserve lives and facilitate the safe return to work and school. Focal points included: laboratory and clinical device evaluation of the limit of viral detection, sensitivity, and specificity of devices in controlled and community settings; regulatory expertise to provide focused attention to barriers to device approval and distribution; usability testing from the perspective of patients and those using the tests to identify and overcome device limitations, and engineering assessment to evaluate robustness of design including human factors, manufacturability, and scalability.

## I. Introduction

The COVID-19 pandemic has presented unprecedented challenges including the critical need to efficiently develop accurate diagnostic tests to rapidly detect symptomatic and asymptomatic infections [1]–[3]. The urgency to develop novel diagnostic devices and scale up existing testing technologies led Congress to appropriate $1.5 billion to the National Institutes of Health (NIH) which, in turn, established the Rapid Acceleration of Diagnostics (RADx^SM^) initiative with the overall goal to significantly expand the ability to mass screen Americans to preserve lives and facilitate the safe return to work and school [1]. RADx Tech was born out of this program with the specific charge to identify, accelerate the development of, scale up, and deploy innovative point-of-care technologies. To meet the critical need for test verification and clinical studies of novel SARS-CoV-2 diagnostics within RADx Tech, a pre-existing set of Point-of-Care Technology Research Network (POCTRN) development centers across the United States were pivoted to provide this critical infrastructure, knowledge, and expertise.

Already well established prior to the pandemic, POCTRN uses a partnership model to improve clinical care through exploratory development of point-of-care test (POCT) devices, clinical needs assessment, training of technology developers, clinical testing, and the provision of administrative support [4]–[6]. The device verification needs for RADx Tech were quickly met by leveraging the academic and medical partners within one of the National Institute of Biomedical Imaging and Bioengineering (NIBIB)’s funded POCTRN centers, the Atlanta Center for Microsystems Engineered Point-of-Care Technologies (ACME POCT) [7]. Established in 2018, ACME POCT is a partnership between Emory University (Emory), Georgia Institute of Technology (GT), and Children’s Healthcare of Atlanta (Children’s). Another POCTRN center, the Center for Advancing Point of Care Devices in Heart, Lung, Blood, and Sleep Disorders (CAPCaT), funded by the National Heart, Lung, and Blood Institute (NHLBI), was enlisted as a secondary and complementary test verification site and was engaged on an as-needed basis. CAPCaT, also established in 2018, is a partnership between the University of Massachusetts Medical School (UMMS) and the University of Massachusetts Lowell (UML).

**Figure 1.**
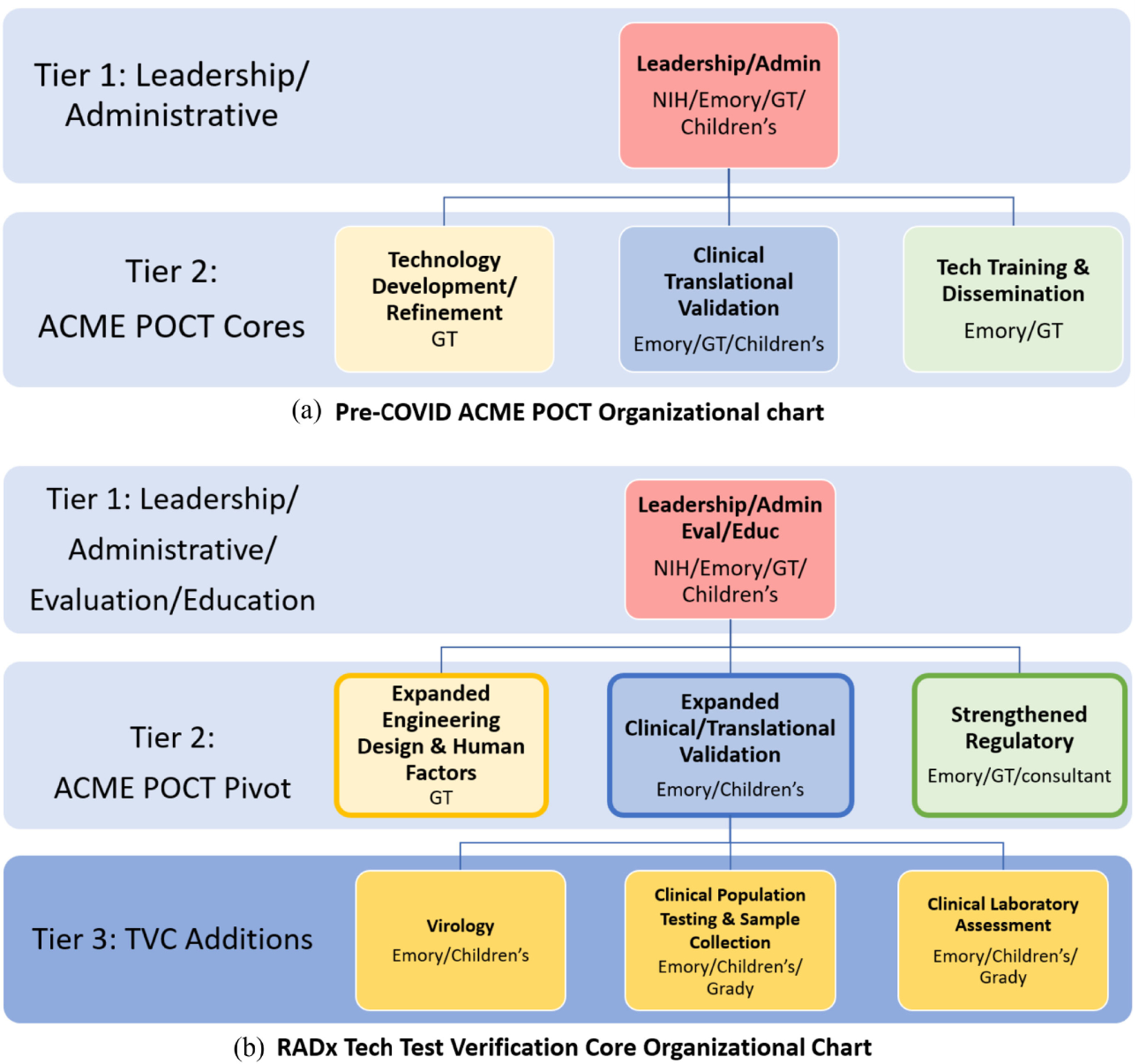
(a). Original organization of ACME POCT. Launched in Sept 2018, the ACME POCT funded by grant U54EB027690 was established to enable inventors with their microsystems-based POC technologies for cardiac, pulmonary hematologic and sleep applications by assisting in defining specific clinic need, conducting clinic validation, and refining these technologies, with the objective of accelerating the path to translation and clinic adoption. (b). High level organizational chart for the newly formed TVC multi-disciplinary and multi-institutional team. Tiers 1 and 2 as shown were largely established part of ACME POCT and quickly pivoted to address the goals of the RADx TVC. Additionally, Tier 3 was assembled to address new areas of need. Evaluation and education function from the tech Training & Dissemination core were moved to the Tier 1 oversight function and the regulatory function was strengthened considerably within the Tier 2 Team. Emory, Emory University and Emory Healthcare; GT, Georgia Institute of Technology; Children’s, Children’s Healthcare of Atlanta; Grady, Grady Hospital.

Fig. 1 illustrates how the original NIBIB-funded ACME POCT organization (Fig. 1(a)) was leveraged to stand up the new “Test Verification Core” (TVC) supplement for this emergency purpose (Fig. 1(b)). The TVC was tasked with serving as the national test verification hub for RADx Tech by providing independent and impartial assessment of the design and performance of promising COVID-19 diagnostic tests developed by private companies and academic inventors [8].

To provide this important “testing of the tests,” the TVC had to quickly assemble a multi-institutional, transdisciplinary team that included the following major focal points for expertise and activities: laboratory and clinical device evaluation to understand the limit of viral detection, sensitivity, and specificity of candidate devices in controlled and community settings; strengthened regulatory expertise to provide more focused attention to identify and overcome barriers to device approval and distribution; usability testing from the perspective of patients and those using the tests to identify and overcome device limitations, and engineering assessment to evaluate robustness of design including human factors, manufacturability, and scalability.

The already established ACME POCT (Fig. 1(a)) was well positioned to assume the TVC responsibility, as the pre-COVID goal of the ACME POCT was to assist microsystems-based POC technology inventors from across the country by providing clinical needs assessments, clinical testing and validation, and technology refinement, with the objective of accelerating the path through translation, regulatory compliance, manufacturing, and clinical adoption [7], [9]. Receipt of a supplement from NIBIB in May 2020 launched this new TVC directive by quickly redirecting the existing ACME POCT goals, processes, and personnel to pivot the focus to verification of the performance of COVID-19 diagnostic tests including sensitivity, specificity, limit of detection (LOD), and cross-reactivity with other viruses. The TVC was able to capitalize on the existing ACME POCT expertise and infrastructure and made select strategic additions from the partner sites including Emory, GT, and Children’s. Sites and personnel were quickly organized and mobilized to establish several focused sub cores comprised of biosafety level (BSL) 2 and 3 laboratories, clinical bio-banks of COVID-19 positive or negative patient specimens (including nasopharyngeal, nasal, and saliva samples), community-based collection sites for prospective testing to compare novel diagnostic technologies with the reference method RT-PCR test, and engineering design and human factors assessment labs.

This quickly assembled but carefully structured TVC was organized to provide assessments and consultation on a wide array of diagnostic technologies (*>*50 devices to date) to provide unbiased evaluations of candidate COVID-19 testing devices. The sub cores within the TVC were empowered to conduct independent evaluations of the devices that were delivered to the TVC. These reviews were then collated by the team evaluators and collectively reviewed by the TVC team using NIH study section type reviews. An impact score was generated and a formal evaluation report was submitted to the NIBIB. The TVC evaluations were factored into the NIH go/no-go decisions regarding further support for testing and manufacturing of those technologies towards making them rapidly available to the public. This article provides a description of the formation and operations of the TVC, elucidates center processes and efforts, and illustrates how a center can provide a model of vision, leadership, coordination, and services to develop and evaluate novel COVID-19 testing devices and thus contribute to the national effort to improve large scale diagnostic testing efforts.

Device verification requires a wide variety of expertise and technical skillsets, and to be done well, must be conducted in a coordinated and integrated manner that is highly dependent upon strong communication and collaboration [4], [7]. Below are descriptions of the key functions of the TVC including 1) Leadership & Administrative including evaluation and education functions; 2) Engineering Design and Human Factors; 3) Clinical/Translational Validation including virology, clinical population testing and sample collection and clinical laboratory assessment; 4) Regulatory. While these sub cores were staffed to function as independent units, strong leadership oversight and a carefully designed communication strategy were implemented to coordinate and integrate their activities to generate a well-informed, unified, multi-disciplinary, expert recommendation for each device that entered the TVC.

Through a $20M supplement, a similar but smaller scale TVC was organized at CAPCaT. They also were able to pivot their pre-COVID 19 activities to assist RADx Tech with the verification of the performance of COVID-19 diagnostic tests including sensitivity, specificity, limit of detection (LOD), and cross-reactivity with other viruses. Because CAPCaT lacks a BSL-3 facility for direct virology testing, however, the ACME POCT served as the primary TVC site calling on CAPCaT for additional testing efforts. The verification operations at CAPCaT were housed at the Massachusetts Medical Device Development Center (M2D2). M2D2 is an incubator program for early-stage medical device and biotech startups, a joint program between University of Massachusetts Lowell and University of Massachusetts Medical School. M2D2 operates three lab facilities; two on the UMass Lowell campus in Lowell, Massachusetts and one on the UMass Medical School campus in Worcester, Massachusetts.

## II. Leadership & administrative including Evaluation and Education Functions

The “team science” literature routinely indicates that progress in scientific research benefits from diverse sources of ideas, innovation, and interdisciplinarity [10]–[12]. Quickly developing a TVC team structure and communication strategy that could develop efficient and effective pathways to facilitate comprehensive evaluation of a largely heterogeneous set of COVID-19 testing devices required experts from different disciplinary backgrounds to work collaboratively and across research area boundaries to synthesize their unique expertise, institutional resources and strengths, and methodologies.

To address the need for a quick launch of this team science approach, the existing ACME POCT leaders and key personnel worked together to address the new TVC priorities by pivoting leadership focus and activities, adjusting goals related to this new urgency and establishing an associated organizational structure and aligned communication strategy. The ACME POCT leadership already in place (Fig. 1(a)) included experts in engineering, clinical and clinical need assessment, regulatory, evaluation and biostatistical, educational, and administrative domains. Key personnel from the existing ACME POCT team as shown in Fig. 1(a) assessed the current area strengths and compared them to the TVC specific needs to focus efforts on identifying and recruiting the additional skillsets and expertise needed to address the unique TVC aims. In response, the ACME POCT leaders identified and recruited resources and targeted expertise throughout the partner institutions in virology, microbiology, laboratory diagnostics, biological specimen collection, clinical research, and biorepositories to join the team. The team grew from nine core ACME POCT individuals to a team of over 100 people in the TVC, many of whom had never worked together. Once these human resources were identified, it was imperative to create a culture of teamwork, collaboration, and streamlined communication with this newly formed, expanded multi-institutional, and interdisciplinary team. A purposeful communications strategy was also established to ensure effective cross-communication among all areas. This included reliance on Zoom for regular video-conference meetings internally and externally with companies and the NIH and use of the Microsoft Teams collaboration and project management platform to facilitate strong and effective local project management practices. A key factor during this initial launch was the engagement of two Chief Operating Officers (Co-COOs) who provided consistency and served as central points of communication through attending all sub core, NIH, and project team meetings to ensure the aims and goals of RADx Tech were articulated clearly. The Co-COOs ensured all personnel had consistent instructions and clear communication on direction and goals to generate the data and input necessary to quickly yet thoroughly evaluate each candidate device. Additionally, the Co-COOs participated in the weekly RADx Tech project report-out meetings, providing real-time project-specific updates to RADx Tech leadership teams and NIH leadership, as well as sharing timely feedback to the TVC on the status of individual projects. Importantly, the Co-COOs were existing employees at Emory with strong ties at the partner institutions and hence, had a familiarity with the people and systems thus allowing them to effectively and rapidly promote a team culture and facilitate effective communications.

The TVC began by having weekly meetings with each of the sub cores (virology, laboratory validation, engineering, clinical site and specimen related) that were focused on setting up the infrastructure, gaining consensus on testing protocols, establishing a useful workflow and communication strategy, and determining which sites and labs would be involved in each project. As time went on and each sub core was established more fully, many of these meetings evolved into twice weekly “all cores” meetings, focused on TVC-wide operational discussions and study sections whereby discussions would focus on recently tested technologies and the data generated from such, additional NIH requests for timely input from experts at the TVC, and other topics relevant to multiple TVC sub cores. Routinely 40+ individuals participated in the TVC all cores meetings, with multiple representatives from each sub core (see Fig. 1(b)) in attendance. The various disciplines represented provided a wealth of knowledge and expertise to refine and expand the TVC’s approach to testing the novel diagnostics on behalf of the NIH.

Importantly, leadership representatives from the institutional Offices of Research Administration were also heavily involved and invested in the successful TVC launch and were devoted to providing ongoing careful oversight. At the start, weekly meetings with the Emory Vice President for Research Administration, departmental leaders, and leaders from key operational offices including sponsored programs, technology transfer, research administration, environmental health and safety, and the Institutional Review Board (IRB) were convened with TVC leaders and the Co-COOs to enable quick attention and effective remedies to address barriers and challenges as they arose. These meetings laid the foundation for streamlining of processes and evaluations – such as template agreements that could be used with RADx Tech companies – and served as a forum to determine how best to address the complexities and challenges with some of the companies. Dedicated attention, collaboration and support from administrative leadership at all the partner institutions (Emory, GT, Children’s) helped tremendously with multiple challenges such as identifying available and qualified research personnel, expediting Material Transfer and Data Use Agreements, IRB amendments, and troubleshooting subcontracting issues just to name a few.

## III. Engineering Design and Human Factors Sub Core

The original ACME POCT Technology Development & Refinement core was already staffed with a multi-disciplinary team of skilled engineering staff and faculty based at GT and Emory. The existing technical expertise of the team (chemistry, physics; and mechanical, electrical, and biomedical engineering) allowed for consideration of the challenges presented by novel SARS-CoV-2 tests and included individuals with experience in fluid mechanics and microfluidics, point-of-care diagnostics, medical devices, micro-electromechanical systems, optical sensors, device usability and human factor design. Usability, design, and human factors expertise was added to the engineering sub core team to effectively pivot and comprehensively meet the TVC needs.

The engineering core performed three essential functions within the TVC. First, when a technology was introduced into the core workflow, initial evaluation of the maturity of the technology within the context of technology readiness levels was established using a benchmarking method for assessing the maturity of acquired technologies [13]. Using this evaluation framework, the maturity of incoming technologies was rated on a 9-point scale ranging from technologies in the idea phase, through prototyping, validation, and production [14]. Many technologies that came through the engineering core were pre-production systems that had been or were currently being validated in the intended operating environment. This maturity assessment provided insight to TVC and RADx Tech leadership into how much work from a technical perspective would be required for each technology to reach commercialization, providing a roadmap of the steps necessary to reach a state suitable for consumer use.

Second, after assessing the maturity of a technology, the engineering core provided a thorough analysis of device design and function. Sometimes preceded by technical discussions with company staff and NIH project leadership, this analysis typically involved device disassembly and a detailed assessment of individual components within the technology. This assessment allowed the Engineering Design and Human Factors sub core to provide feedback on potential failure modes and engineering best practices, in turn allowing the overall team to understand reasons for technology failures, to 1) help the companies iteratively improve their technologies, and 2) assist the laboratory and clinical-based sub core teams with test operation. Among the challenges of this assessment were the diversity of sample types (swabs, saliva, breath) and sampling methods, finding suitable lab space for device disassembly and analysis in this new remote-work environment, test target molecules (RNA or protein antigen), and signal transduction mechanisms (electronic or optical, and visual or non –visual). Furthermore, given significant concern of SARS-CoV-2 exposure to lab personnel involved in testing and patients when interacting with these devices, the Engineering Design & Human Factors sub core performed a biosafety analysis to identify potential sources of device contamination and suggested mitigation practices. This critical role of this sub core allowed for proactive adoption of careful use and practice to avoid biosafety issues and protect test validation personnel as well as our patient volunteers.

Finally, the Engineering Design & Human Factors sub core analyzed materials and manufacturing choices that went into device design using both information provided by the company and physical analysis of the technology. The engineering core communicated these findings directly to the RADx Tech large-scale commercialization core that had been established by the NIH to facilitate the manufacturing scale up of successful technologies (see article by Walsh et al. in this special issue). This analysis provided this group a “head start” so they were prepared for the challenges that needed to be overcome for manufacturing and scalability of these technologies, and could begin to address these challenges prior to receiving the technology [15].

Device usability testing aligned with human factors was performed by HomeLab, a multi-disciplinary team of faculty and staff based at GT who focus on user experience research, human factors, industrial design, and mechanical and electrical engineering [16]. HomeLab allowed for a thorough evaluation of device usability considerations for multiple user groups, the assessment of viability of potential use cases, and the development of objective recommendations for device improvement to be relayed to project teams.

Technologies were evaluated to determine the viability for the intended (and potential) use cases using a variety of methods, depending on factors such as timeline, target end user, and device development stage. Evaluation methods included expert reviews, in-depth expert evaluations, design failure modes and effects analyses, heuristic analyses, use case simulations, user observations, and user testing. Challenges to device evaluation included short timelines, limited numbers of devices to assess, and communication of technical assessments to the larger center. Each usability evaluation had to be tailored to the technology and individual circumstances. For a device that was early in the design cycle, focus was placed on providing actionable feedback that could be rapidly incorporated into the design changes. For a device that was further along in the design process, emphasis was placed on details of the product design or recommendations for the instructions for use that would result in reduced risk or enhanced user satisfaction.

The engineering research team also provided recommendations to improve the design of the protocol, device, and/or instruction materials through a comprehensive usability report that included results of human factors analyses, results of simulations and accessibility measurements, user feedback, and a comprehensive rating scale that contributed to informed TVC reviews. Some common examples of recommendations include increased font size and reorganization of the instruction materials to increase accessibility and comprehension for the user, as well as physical adjustments to the devices to allow users with reduced dexterity to better use the device. The engineering team also used common convention and design principles to make recommendations on what would be most intuitive to users to decrease frustration and reduce the risk of errors. The device development stage was factored into recommendations, with emphasis placed on recommendations that would be feasible to implement in a short time period.

The TVC at CAPCaT also supported the advancements of several different technologies that went through the standard RADx Tech review process by standard validation and usability testing. CAPCaT contributed through development of new lab experimental setups to mimic physiological relevant conditions in a laboratory setting, such as with a nasal tissue model to test and refine new swab designs in the pre-clinical stage of an injection molded swab.

## IV. Clinical Translational Validation

The original ACME POCT Clinical and Translational Validation Core experienced the most expansion and specialization upon the launch of the TVC. The preexisting ACME POCT clinical leaders were instrumental in identifying, attracting and recruiting the additional expertise necessary for the specialized evaluation of the COVID-19 detection process. The expansion was deemed necessary in three key areas as described below.

### A. Virology Sub Core

Immediately upon the TVC launch, a critical need was identified for testing live virus under controlled laboratory conditions to enable thorough device testing. While interrogating human samples from COVID-19 infected patients would undoubtedly play a central role in the evaluation process, use of live virus allowed for the generation of data under carefully controlled conditions to report accurate sensitivity and specificity results reflecting utility of these technologies designed to detect SARS-CoV-2 antigens and/or viral nucleic acids. Existing access to a biosafety level-3 (BSL-3) laboratory and already trained personnel with decades of experience with BSL-3 standard operating procedures were instrumental in enabling the ACME POCT TVC to provide this indispensable device verification platform. This was accomplished via collaboration and combined contributions from two existing Emory-based laboratories, one in the Laboratory of Biochemical Pharmacology and the other within the Division of Pediatric Infectious Disease. These labs were staffed by a combination of faculty and staff who shared protocols and resources, split tasks based upon staff availability. Personnel were responsible for preparing virus stocks, troubleshooting tests, writing reports, meeting with project teams and their NIH liaisons and discussing new projects and identifying critical new collaborations. While the two labs provided some level of redundancy to allow for testing of multiple devices at one time, they also provided a level of specialization due to their access to and use of different virus strains. Specifically, one lab used USA-WA1/2020 and Australian strain CoV/Victoria/1/2020 and the other lab used USA-CA3/2020, Italy-INMI1, and USA-GA4/2020. The latter strain is a virus isolate sourced from a locally hospitalized patient that was used to expand the rigor and significance of our device testing protocols. All isolates were propagated at low passage levels in our laboratories and titration was performed using the TCID50 method. Additional specialized expertise at Emory was used to perform viral sequencing and PCR quantification of the produced stocks. Overall, these collective arrangements allowed for a very functional, efficient, and customizable way to thoroughly evaluate devices using live virus, and moreover highlights our ability to capitalize on area strengths to provide comprehensive and rigorous testing approaches.

The Virology sub core experts also served as functional partners to the private companies and academic inventors and/or NIH Team Leads, through their preparation of LOD testing protocols, LOD testing, and reporting of the operational performance of the evaluated tests (LOD and practicality). The LOD of the tests was objectively determined by using serial dilutions of live SARS-CoV-2 of known concentrations in relevant pooled negative human samples including saliva and nasal swab matrixes. This sub core was responsible for obtaining and propagating emergent SARS-CoV-2 isolates/variants to expand the testing capabilities as previously described.

The creation of this sub core initially faced several challenges including (i) a need to rapidly recruiting additional qualified personnel and training them for BSL-3 work, (ii) PPE and disinfectant shortage (iii) the need for rapid implementation of new Biosafety SOPs and obtaining their approval by the Environmental Health and Safety Office (EHSO) in the context of evolving safety guidelines and regulations (iv) limited access to reagents such as the viral isolates and (v) new equipment requirements. These challenges were addressed in a timely manner in large part due to the aforementioned institutional leadership focus and partnership. For example, despite an Emory-wide hiring freeze, this sub core was granted specialized exceptions to quickly post job openings, recruit, and hire to meet critical personnel needs.

### B. Clinical Population Testing & Sample Collection Sub Core

This sub core included adult and pediatric arms, and each group had two main aims. The first aim was to conduct in-vivo testing to determine the performance characteristics of test platforms by testing directly in the patient population, and the second was to amass a collection of a variety of human samples including nasopharyngeal swabs, nasal swabs, oropharyngeal swabs, and saliva samples to bank for future *ex-vivo* testing. These aims were largely accomplished via four sites including 1) a Children’s sponsored pediatric drive-thru testing site; 2) an Emory-based adult stand-up site created specifically for this purpose; 3) a Grady Memorial Hospital-based site dedicated to healthcare staff and inpatient testing; and 4) both hospital-based and Emergency Department recruitment from Children’s, Emory (both Emory University Hospital and Emory University Hospital Midtown) and Grady Memorial Hospital. These sites were staffed by clinical research coordinators and clinical research nurses with oversight by faculty researchers who were responsible for training, consenting, data collection and direct device testing [8].

Upon TVC launch, this sub core had to quickly identify suitable sites that produced the necessary variety in patient populations to meet potential testing needs. This included asymptomatic and symptomatic adults and children in different areas of metro Atlanta while maintaining proximity to the applicable biorepository. The Children’s drive-thru was an existing site operationalized on May 11, 2020, to meet the testing needs of the pediatric population but had been scheduled to dissolve July 1, 2020. The sub core capitalized on the opportunity to keep that facility operational for the duration of TVC. This extension of operations provided a win-win outcome and is a good example of successfully capitalizing on a synergy between clinical and research operations. The Children’s drive-through was the first established clinical site for the TVC and recruited over 3000 children between June 2020 and January 2021. Establishment of the adult research sites required additional negotiations between research and clinical healthcare teams to incorporate the research protocols alongside the established clinical operations. Success grew from a close partnership including the engagement of the onsite clinical managers at each adult site to ensure a smooth working relationship and establishing an atmosphere to allow both sides to articulate their individual and collective needs to operate harmoniously while still meeting individual goals. Ultimately, recruitment was accomplished from all established sites with little disruption to the clinical testing workflows, which was the most common obstacle.

The creation of the TVC biorepository was also an early activity. The Department of Pediatrics had a preexisting research biorepository which rapidly adapted existing processes for sample receipt, processing and storage, inventory management and sample distribution to internal and external labs. Some customization was necessary including coordinating courier services to new and multiple locations, protocol development for new sample type processing and storage, customizing and linking laboratory inventory management systems with the clinical database, and diversion of effort and training new personnel to meet the scope and scale of the project. The relatively small and already established pediatric biorepository operation was able to nimbly and successfully pivot and expand to accommodate the TVC needs. The pediatrics personnel then provided consultation and input to adapt their processes and share their experiences with their adult counterparts so that they too could establish a parallel adult-focused sample biorepository. The CAPCaT TVC also provided access to SARS-CoV-2 clinical samples through a large, existing biorepository at UMMS, which has an established relationship with the Department of Pathology in the affiliated University of Massachusetts Medical Center, Worcester, MA. In May 2020, the UMMS biorepository adapted its procedures to collect, process and store remnant clinical human samples from the clinical system, including nasopharyngeal swabs, saliva, serum, and plasma. The swabs and saliva samples were assayed by RT-PCR using the TaqPath COVID-19 Combo Kit (Applied Biosystems).

This sub core also played an essential role in verifying and collecting clinical data for RADx Tech-supported companies’ Emergency Use Authorization (EUA) applications. The organization and breadth of our testing sites have allowed for collection of both fresh and banked samples and the ability to query these databases to meet the unique study needs. This sub core has generated important sensitivity and specificity data, as well as practical usability data (produced in collaboration with the aforementioned HomeLab) that has been incorporated in the final TVC recommendation of each device. Feedback from the clinical research nurses and coordinators on instruction for use documents and overall design of some devices during verification have led to improvements and ultimately better products to begin the EUA process.

Several barriers were encountered related to successful recruitment and sample collection that had to be addressed to ensure success within the TVC. The mix of adult and pediatric samples had to be carefully coordinated since the two laboratory information management systems (LIMS) were not linked, due to pediatric and adult operations using different vendors for their electronic medical record systems and consistent records were needed to comply with FDA part 11 research record keeping compliance. Another challenge experienced was when companies requested biorepository samples be shipped to them so that they could do their own testing rather than send their device to the TVC. Especially early on, there were very few banked samples available for testing off site so TVC Leadership was tasked with making decisions when a company’s request for scarce *ex vivo* samples could be accommodated without compromising on-site TVC operations and testing.

Given the variety and capacity of the clinical sites, this team was able to pivot quickly to coordinate and test multiple devices at a time when the number of devices entering the TVC workflow increased. The clinical team worked with the Co-COOs to match the devices to the best sites and to coordinate the time the testing for each one based on current volume and positivity rates. Communication was key to avoid straining the team while also capitalizing on the full system capacity. Unique devices that required specialized sample types not yet encountered, such as taking measurements from exhaled breath, presented challenges that were addressed through our diverse team-based approach. The unique requirements presented in these situations required collaboration among all the sub cores to determine safety and usability with a major focus on ensuring there would be no transmission of virus between patients and testers. In this example, to ensure a thorough assessment, input from clinical experts in the pulmonology field was sought utilizing their established best practices with forced expiratory volume tests and the associated cleaning practices to ensure our usability test protocols were conducted with the highest safety standards across all sub cores and clinical population testing sites.

### C. Clinical Laboratory Assessment Sub Core

The Clinical Laboratory Assessment sub core had three aims. The first was to perform reference SARS-CoV-2 testing on samples collected from the research participants recruited at the clinical population testing sites described above. This reference result was not only used as a comparator for device testing, but also as a comparator for patient specimens saved in the biorepository. The second was to “test the test.” This consisted of evaluating the numerous devices submitted to the TVC to help inform the recommendation regarding the clinical utility and value of the device. The third purpose was to consult with industry and the inventors upon request to discuss their devices and advise the manufacturers on usability parameters to improve.

Four existing laboratory sites were leveraged for this work – 1) the Advanced Diagnostics Laboratory in the Children’s clinical lab; 2) the Emory/Children’s Laboratory for Innovative Assay Development (ELIAD); 3) the Emory Investigational Clinical Microbiology Core; and 4) the Grady Memorial Hospital Clinical Microbiology Laboratory. At the launch of the TVC, these sites were already staffed by licensed medical technologists, research technicians, and post-doctoral fellows although the volume of work from RADx Tech necessitated recruitment of additional staff to assure rapid turn-around time.

Within these laboratories, there were several platforms for routine clinical SARS-CoV-2 testing by RT-PCR using assays that had received EUA and that can be used as comparators. The Children’s site acquired a Panther Fusion (Hologic, Marlborough, MA) instrument and a Liaison (Diasorin, Cypress, CA) for their SARS-CoV-2 reference testing. The ELIAD site referred molecular testing to Emory Medical Laboratories or the other sites. The Investigational Clinical Microbiology Core developed a multiplex SARS-VoV-2 real-time RT PCT assay that was validated against FDA authorized diagnostics [17]. This research-use assay provided a flexible method for use with a variety of specimen types and perhaps even more importantly, could be modified to detect emerging variants. The Grady Clinical Microbiology Laboratory used the Alinity M (Abbott Molecular, Des Plaines, IL). While there was initial discussion about standardizing reference testing across sites, the group ultimately decided to follow protocols at their individual sites, allowing a breadth of assessment parameters and a “divide and conquer” capability when multiple devices arrived at the same time for evaluation.

Each site performed a sequential series of analyses to evaluate the performance of tests submitted to the ACME POCT TVC. In many cases, these evaluations included the use of contrived and coded panels to determine analytic sensitivity (LOD) and specificity (using the OC43 and MERS coronaviruses), before testing with authentic clinical samples. The workflow at all Clinical Laboratory Assessment sub core sites was multi-fold. Before a device was scheduled for delivery to the TVC, key representative members of this sub core met with the company or assay development team by Zoom to discuss the principles of the assay, existing data on assay performance, and other specifics which might affect the performance testing. This information was used to select the form of inactivated SARS-CoV-2 used in testing (assays designed to detect viral antigen were typically evaluated using gamma-irradiated virus, while heat-inactivated virus was used when evaluating RNA detection assays), the matrix (saline, UTM, VTM, saliva, and serum), and the specific dilution range to focus on testing. The next steps consisted of a team effort of creating the testing panels, sample coding, performance testing, recording results, and then finally unblinding the samples to collate the results.

While this was the typical pattern for testing, at times customization of testing protocols was necessary. For example, some devices were too large or the technology was not sufficiently mature to ship to the TVC. In these cases, this sub core prepared and coded assay panels as described above, and then shipped the panels to the companies for testing. Additionally, the TVC was occasionally asked to evaluate an assay where the devices could be shipped, but a company representative visited on site to assist with some of the challenges of operating the instruments. In these cases, a standard approach was used, making sure all the individuals involved in testing were blinded to sample identifiers.

While some assays performed considerably below acceptable specifications, many others yielded promising results. This raised the question of how to compare the performance of the better assays, since they were often very different technologies. One approach to addressing this problem was to collect SARS-CoV-2-positive nasopharyngeal swabs from the participating biorepositories, pool the samples, and then freeze the resulting pool in single-use aliquots. This approach eliminated several problems: the results were not affected by inactivation methods, the matrix was one of the most commonly used for diagnostics, the exact same sample could be used for several different devices, the testing could be performed in the BSL-2 enhanced laboratory, and the aliquots were sufficiently concentrated that several 10-log dilutions could be performed to objectively compare sensitivity of assays to each other.

At the end of the testing phase, detailed performance reports were prepared by each laboratory site and the data were collated along with the data generated by the Virology sub core. A comprehensive data assessment was facilitated by the Clinical Laboratory Assessment evaluation team to determine if the test performance justified further testing using authentic clinical samples. That testing was initiated if the initial performance LOD was sufficiently good.

There were multiple barriers in scaling up a clinical laboratory to meet the new demands of the TVC, especially during an already resource-constrained pandemic. Barriers included reduced availability of instruments to purchase, which necessitated forging a close relationship with the vendor who supplied instruments as soon as they were available. There were also significant issues with availability of supplies including shortages of PPE, pipette tips, gloves, transport media, and collection swabs. Thanks to the national RADx Tech infrastructure, the large-scale commercialization core was able to help acquire scarce supplies. Access to qualified laboratory personnel was also an initial constraint. Although at the TVC launch there were well qualified staff in place, the volume of the TVC work required the addition of skilled laboratory personnel to accommodate the pace and workload. The central Emory Human Resources office was enlisted to in part to help address this by providing lists of personnel from labs whose research had ramped down at the beginning of the pandemic. In fact, this same principle was used to quickly meet many of the workforce needs across the TVC. In this particular case, since the clinical volumes for routine clinical testing decreased during the pandemic because of decreased patient volume, it was possible to utilize technical and management personnel placed on part-time furlough. This allowed for technical support that knew the details of test validation, and who also knew how to “get things done” in a hospital system to become part of the TVC team. The doctoral staff, while fully engaged with daily duties, were flexible with their work to accommodate the multiple meetings and testing of the devices. Overall, a commitment from Children’s and Emory research, purchasing, finance, and administrative teams was instrumental in supporting the TVC initiative.

## V. Regulatory Sub Core

The need for regulatory expertise to assist technology developers to navigate the Emergency Use Authorization process became increasingly vital as the COVID-19 pandemic unfolded and it was clear that an accelerated timeline of FDA approval was needed. Furthermore, as the RADx Tech portfolio of candidate devices grew and diversified and guidance from FDA was developed and evolved, project teams were in great need of continued regulatory consultation. Early in the TVC initiative, it was clear that project teams would need to compile and organize regulatory resources as rapidly as possible. As such, the ACME POCT greatly expanded its pre-existing regulatory team and assigned a dedicated operations lead and facilitator. Together, the team connected with regulatory experts in other RADx Tech centers to create a unified effort to address the needs of the growing number of projects.

The regulatory sub core consisted of four team members from GT, Emory, and an outside consulting firm with experience in regulatory operations and coordination. Team members were assigned to specified RADx Tech projects to provide customized support per unique project and its technology needs. Examples of customized support included regulatory strategies, frequent project consultations, drafting and/or review of: Pre-EUA and EUA submissions, clinical protocols, product labeling, Instructions for Use (IFU), Quick Reference Guide (QRG), and Fact Sheets.

An instrumental element of support can be attributed to a successful working relationship with the FDA. Agency leadership within the Office of In Vitro Diagnostics and Radiological Health worked closely with the Regulatory sub core. Key highlights of this collaboration include 1) Prioritization of RADx Tech Pre-EUA and EUA submissions 2) Weekly status review of RADx Tech submitted Pre-EUA and EUA submissions, 3) Weekly general question and answer meetings 4) Weekly individual project meetings to discuss those assays intended for Point of Care or at-home use and 5) planning for clinical evaluation of new technologies via method comparison studies. Key functions carried out by this sub core included: 1) The creation of regulatory strategies and tools, compilation and submission of EUA with project teams, 2) Organization and facilitation of meetings designating Clinical Laboratory Improvement Amendments (CLIA), Point of Care (POC) & Laboratory Developed Test (LDT), 3) Communication with FDA on pre-EUA and EUA status, 4) collaboration with FDA to determine appropriate clinical evaluation designs and 5) Collaboration with other TVC sub cores to ensure sufficient data to support regulatory packages.

This dedicated sub core team aided in the development of U.S. Regulatory strategies for the teams developing SARS-CoV-2 detection devices. A challenge in this work was the varying levels of experience working with FDA on the part of the private companies and academic inventors. However, this sub core was able to work closely with FDA to recommend standards/consensus standards and guidance documents to support least burdensome regulatory approaches and assisted in the creation of and review of Risk Analyses (in conformance with appropriate guidance/industry and FDA standards, for example ISO 14971) [18], [19]. Specifically, the FDA worked with this sub core to create clinical evaluation protocols by confirming use case (ranging from high and moderate complexity lab use, waived/point-of-care use, and home use), the type(s) of samples to be tested (spanning all upper and lower respiratory specimens and saliva), how many samples, the appropriate comparator assay, number of operators, sites and appropriate acceptance criteria. This led to the execution of successful clinical studies that were used to support EUA, which has enabled several RADx Tech teams to realize their goals and market their finished products in the field.

## VI. TVC Lessons Learned

Reflections and recommendations that have emerged from the TVC include the following (see Table I): 1) Interdisciplinary collaboration among academic researchers, inventors, funders and policy makers, and representatives from clinical users of devices throughout the development, research and decision-making process allows for quick decisions and course corrections, and improves the use of government funds for device development; 2) Discussion of timelines and project deliverables among device developers, researchers and policy-makers enables each group to be more responsive to each other and allows each group to be more aligned; 3) Communicating project plans and data derived from device testing from interdisciplinary contexts is often challenging. Understanding and translating findings in a way that is sufficient to make “go / no go” decisions from one context to another in a quickly moving and audience of multiple stakeholders is critical; 4) A rapidly developing and changing epidemic in which devices are at varying technology readiness levels greatly affects study designs and must be accounted for when planning and evaluating testing devices; 5) Elegant and/or novel device design is not necessarily an indicator of performance, usability, or scalability. Sometimes the most effective test for a new target is the repurposing of existing platforms with proven technology approaches; 6) Academic researchers can improve their work from the inclusion of others that they may see as their competitors (e.g., those who compete for similar funding resources) and those from other disciplinary areas (e.g., engineers, clinical staff, and regulatory specialists) to learn different perspectives and approaches; 7) It is important to continue building US device testing capacity as a means of training future generations of scientists and device developers in order to create an established process and field of accelerated device development in preparation for future public health emergencies (see also article by DiMeo et al. in this special issue).

One of the true assets of the TVC has been the tremendous teamwork and built-in redundancies within some of the infrastructure. With the ebb and flow of when devices were delivered for testing, there were many occasions where multiple assays were being evaluated at the same time. This involved utilization of both BSL-3 laboratories, more than one BSL-2, multiple clinical sites, usability evaluations and the biorepositories. Additionally, the TVC and its involvement of both an adult and pediatric health system ensured that tests were being evaluated with both pediatric and adult patients. Additionally, key to the success of the TVC was the deliberate and strategic partnership that was developed between the project team and federal contacts at NIH and the FDA which facilitated our inter-institutional collaboration, communication, and relationship-building.

## VII. Conclusion

While device test results from this initiative will be published in future papers, the process and experience of launching the TVC has been an instructive experience that warrants sharing to generalize the knowledge gained. There is agreement among the TVC faculty and staff who have worked in RADx Tech that it has been one of the most rewarding projects of our careers. For many of us, we considered this project an honor and our contribution to the worldwide effort to combat the COVID-19 pandemic. Over 100 faculty and staff across our respective institutions participated and the TVC team has been involved in the assessment of >50 different devices, more than 20 of which are now in phase 2 of clinical testing, regulatory approval, and scale up with others already released to the market more likely to follow [20].

The end goal of the RADx Tech was to develop and evaluate novel COVID-19 testing devices and thus contribute to the national effort to improve large scale diagnostic testing efforts. While doing this, the TVC harnessed the best practice of collaboration and communication to quickly develop our team as well as the evidence base for each device. Future efforts will be made to build a sustained collaborative platform for device development using the RADx Tech model beyond the COVID-19 pandemic to facilitate continued accelerated device development for other clinical needs and sharing of lessons learned.

## Figures and Tables

**TABLE I:** Reflections and Recommendations From the Radx Tech Test Verification Core

*Lesson 1 Interdisciplinary collaboration and decision making*	Interdisciplinary collaboration can support quick decisions and course corrections, and can enhance the use of government funds for device development.
*Lesson 2: Discussion of timelines and deliverables*	Discussion of timelines and project deliverables is critical and enables responsiveness and team alignment.
*Lesson 3: Communication and interdisciplinary teams*	Communicating in interdisciplinary contexts is often challenging. The translation of findings to multiple stakeholders is critical to making quick decisions.
*Lesson 4: Technology readiness*	Devices at varying technology readiness levels greatly affects study designs. One size does not fit all and thus, flexible and tailored testing approaches must be used.
*Lesson 5: Device design sophistication*	Elegant and/or novel device design is not necessarily an indicator of performance, usability, or scalability. The full testing battery must be used to make the best and most informed recommendations.
*Lesson 6: Inclusion and teamwork*	Academic researchers can improve their work from the inclusion of “competitors” and those from other disciplinary areas.
*Lesson* 7: *Training for the future*	Future generations of scientists and device developers must be trained in accelerated device development in preparation for future public health emergencies.

## Impact Statement—

This article describes the multi-institutional, transdisciplinary team of the (RADx^SM^) Tech Test Verification Core” and its vital role in expediting evaluations of COVID-19 testing devices.
